# Supplementary material for: Simultaneous profiling of histone modifications and DNA methylation via nanopore sequencing
Source: Nat Commun. 2022 Dec 24;13:7939. doi: 10.1038/s41467-022-35650-2 (PMC9789962; doi:10.1038/s41467-022-35650-2)
Supplement: Supplementary file 1 — Supplementary Information [file 41467_2022_35650_MOESM1_ESM.pdf]

Simultaneous profiling of histone modifications and DNA methylation via nanopore sequencing

Xue Yue, Zhiyuan Xie, Moran Li, Kai Wang, Xiaojing Li, Xiaoqing Zhang, Jian Yan, Yimeng Yin

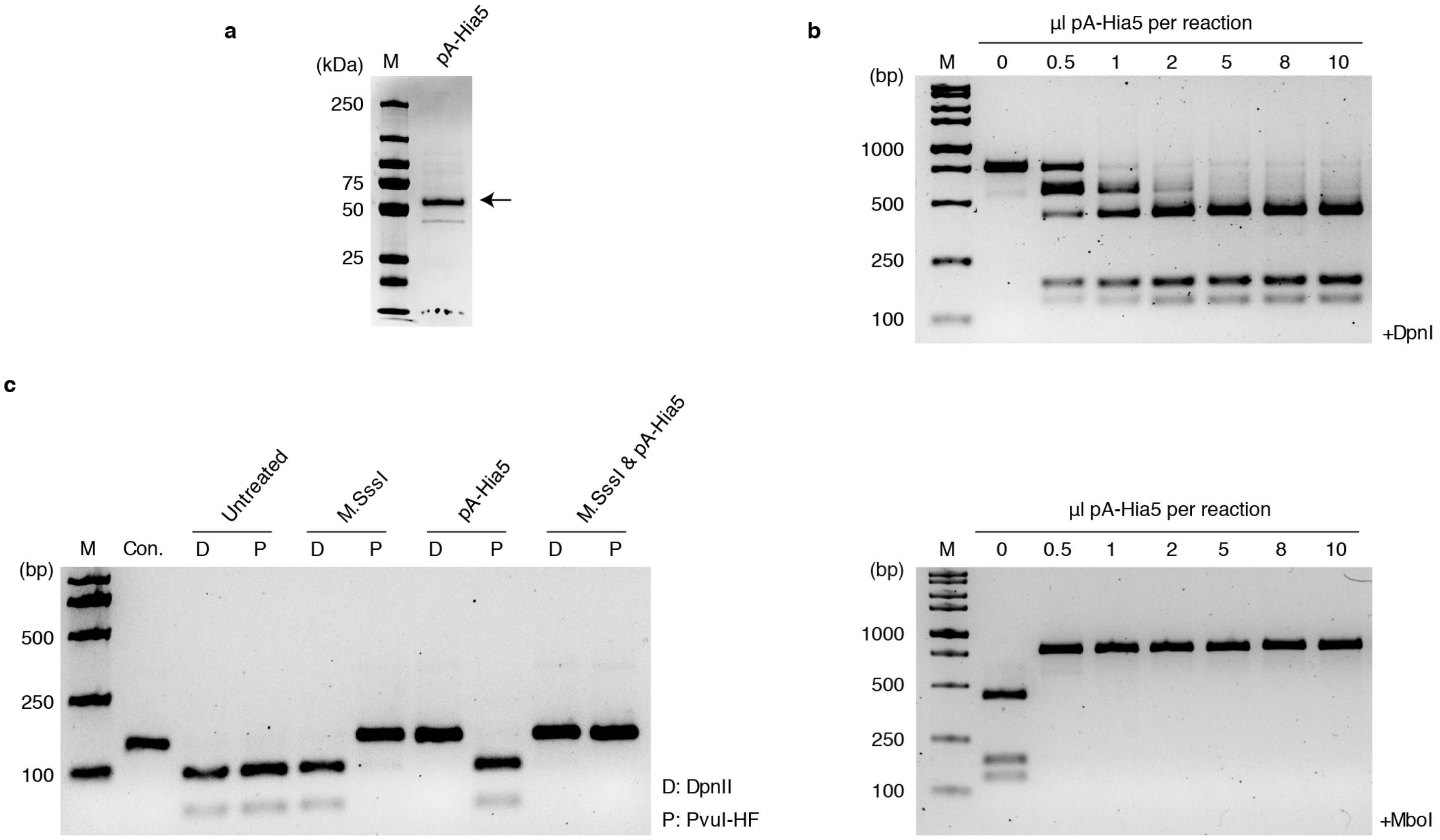

a
(kDa)
M
pA-Hia5
250
75
50
25
b
µl pA-Hia5 per reaction
(bp)
M
0
0.5
1
2
5
8
10
1000
500
250
100
+DpnI
c
Untreated
M.SssI
pA-Hia5
M.SssI & pA-Hia5
M
Con.
D
P
D
P
D
P
D
P
(bp)
500
250
100
D: DpnII
P: PvuI-HF
µl pA-Hia5 per reaction
M
0
0.5
1
2
5
8
10
(bp)
1000
500
250
100
+MboI

Supplementary Figure 1. In vitro assessment of the methyltransferase activity of pA-Hia5. (a) Gel migration showing purified pA-Hia5 protein. The gel was stained by Coomassie Blue and the arrow indicates the band corresponding to pA-Hia5. (b) Testing the activity of purified pA-Hia5. Indicated amounts of pA-Hia5 protein were used to methylate 1μg DNA that contained 4 GATC sites, and the methylated DNA was then subjected to the digestion of the restriction enzyme DpnI and MboI, respectively. DpnI is capable of cutting GATC sites only after the adenine in the sequence is methylated. In contrast, adenine methylation in the sequence GATC blocks the digestion of MboI. (c) Testing the enzymatic activity of pA-Hia5 on DNA with mCpGs. 1μg DNA that contained CGATCG motif was first incubated with M.SssI enzyme to methylate CpG sites and subsequently with pA-Hia5 protein to methylate adenines. The methylated DNA was digested with either DpnII, the restriction enzyme that cuts GATC sites in the absence of 6mA and is insensitive to 5mC, or PvuI-HF, the restriction enzyme that cuts CGATCG sites in the absence of 5mC and is insensitive to 6mA. The DNA treated with either M.SssI or pA-Hia5 was also digested with the two restriction enzymes as control. Three independent biological replicates were performed in (a), (b) and (c). Source data are available in the Source Data file.

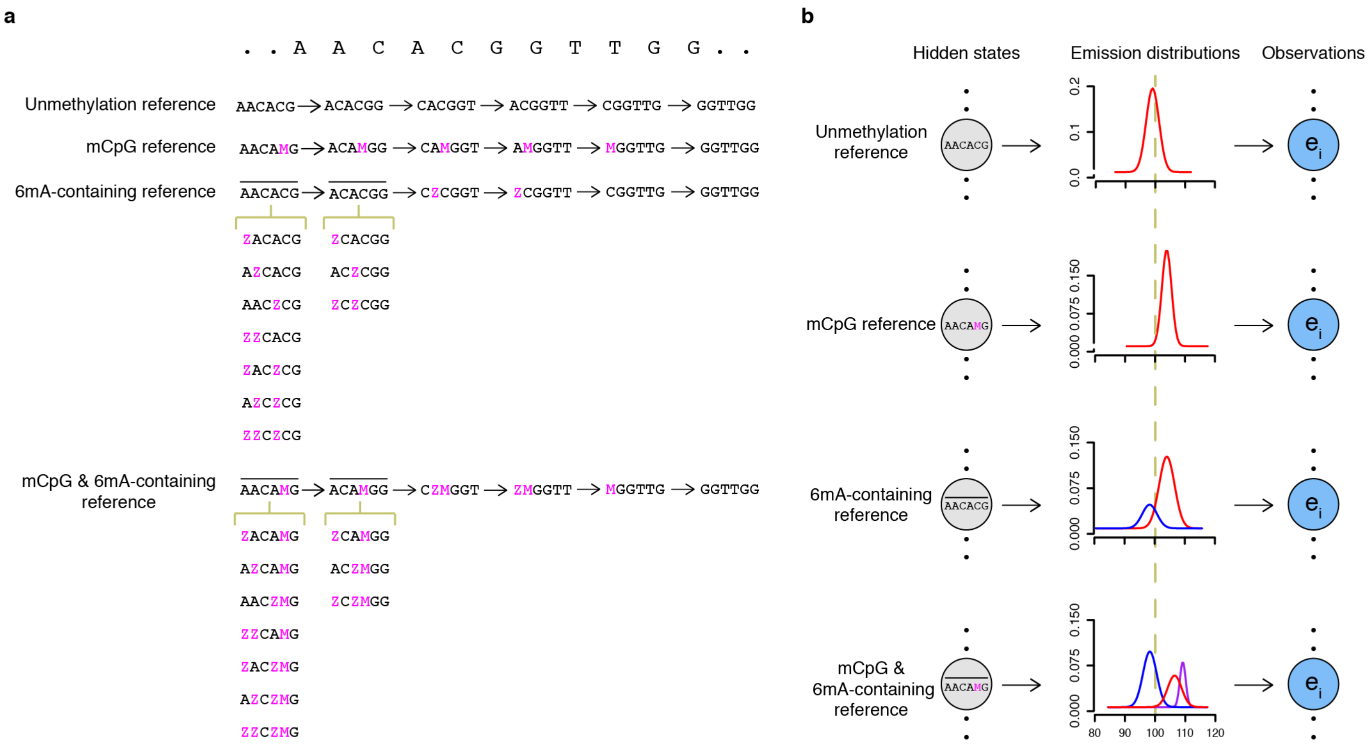
a
. . A A C A C G G T T G G . .
Unmethylation reference
AACACG → ACACGG → CACGGT → ACGGTT → CGGTTG → GGTTGG
mCpG reference
AACAMG → ACAMGG → CAMGGT → AMGGTT → MGGTTG → GGTTGG
6mA-containing reference
AACACG → ACACGG → CZCGGT → ZCGGTT → CGGTTG → GGTTGG
ZACACG
AZCACG
AACZCG
ZZCACG
ZACZCG
AZCZCG
ZZCZCG
ZCACGG
ACZCGG
ZCZCGG
mCpG & 6mA-containing reference
AACAMG → ACAMGG → CZMGGT → ZMGGTT → MGGTTG → GGTTGG
ZACAMG
AZCAMG
AACZMG
ZZCAMG
ZACZMG
AZCZMG
ZZCZMG
ZCAMGG
ACZMGG
ZCZMGG
b
Hidden states
Emission distributions
Observations
Unmethylation reference
AACACG
mCpG reference
AACAMG
6mA-containing reference
AACACG
mCpG & 6mA-containing reference
AACAMG
0.0
0.1
0.2
0.000
0.075
0.150
80
90
100
110
120
e$_i$

Supplementary Figure 2. Schematic of the four datasets for parameters learning.

(a) An example showing four training datasets that contain either unmethylated bases (top), nearly-complete CpG methylation (96.2%; second from the top), partial adenine methylation (second from the bottom) or nearly-complete CpG methylation & partial adenine methylation (bottom). As adenine methylation randomly occurred, a given *k*-mer with more than one adenine, such as AACACG, potentially has methyl-adenine in different position(s). We grouped such *k*-mers as a new *k*-mer $\bar{k}$ in the training datasets with partial adenine methylation and trained one, two or multiple sets of parameters for the grouped *k*-mer $\bar{k}$. (b) An example showing the trained parameters of 6-mers from different training datasets. Note that M represents 5-methylcytosine, Z denotes 6-methyladenine and $\bar{k}$ means a group of *k*-mers that contain methylated adenine(s) in different positions and share the same nucleotide bases composition and order except A/Z. Source data are available in the GitHub https://github.com/YinLabTJ/nanoHiMe.

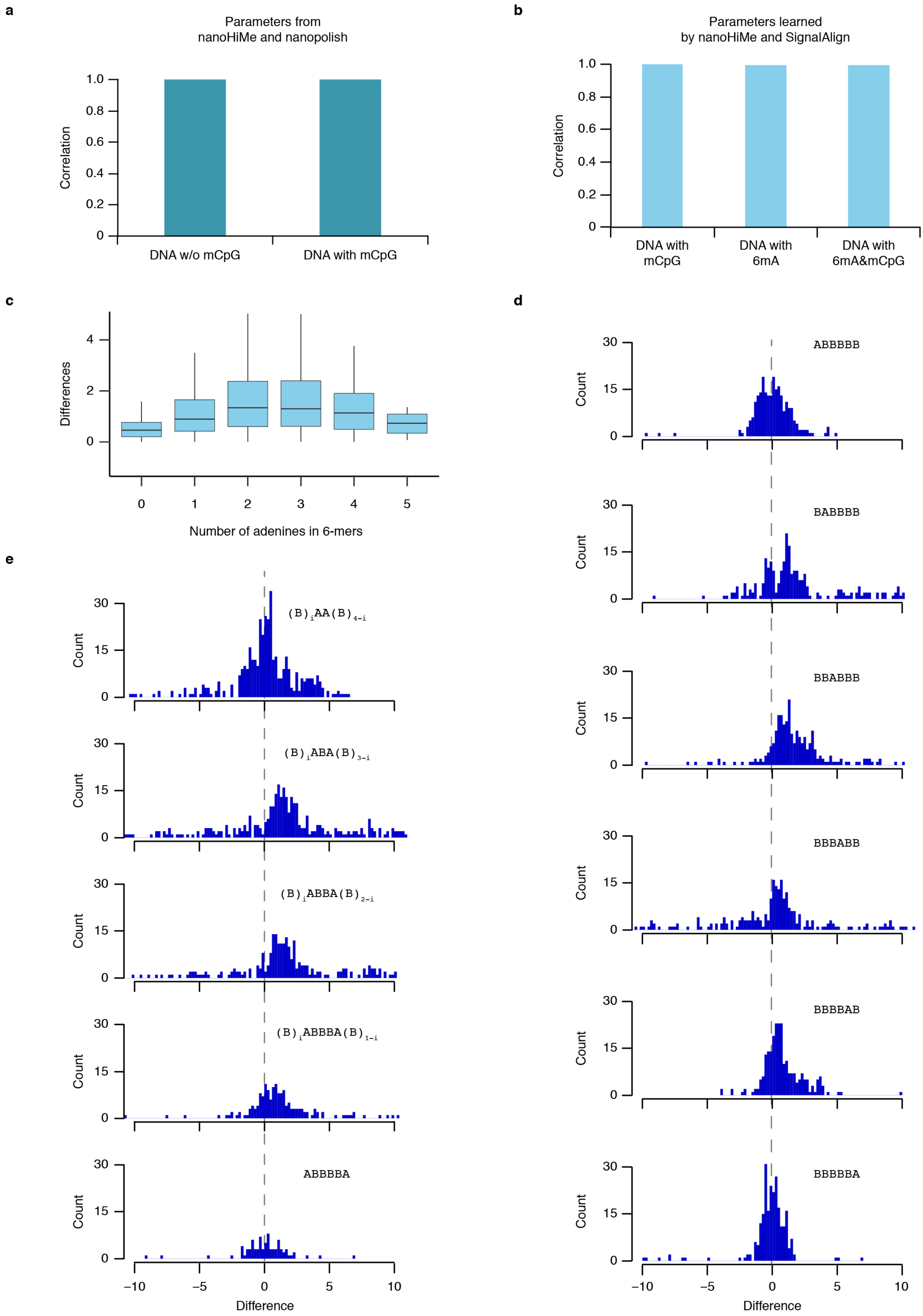

a
Parameters from
nanoHiMe and nanopolish
Correlation
1.0
0.8
0.6
0.4
0.2
0
DNA w/o mCpG
DNA with mCpG
b
Parameters learned
by nanoHiMe and SignalAlign
Correlation
DNA with mCpG
DNA with 6mA
DNA with 6mA&mCpG
c
Differences
Number of adenines in 6-mers
d
ABBBBB
BABBBB
BBABBB
BBBABB
BBBBAB
BBBBBA
Count
Difference
e
(B)iAA(B)4−i
(B)iABA(B)3−i
(B)iABBA(B)2−i
(B)iABBBA(B)1−i
ABBBBA
Count
Difference

Supplementary Figure 3. Assessment of the learned parameters and the effect of adenine methylation on the parameter's shifts.

(a) The Pearson correlation coefficients showing the comparison between the parameters provided by nanopolish and those learned by nanoHiMe from PCR amplicons without treatment or treated with M.SssI. (b) The Pearson correlation coefficients showing the comparison between the parameters learned by nanoHiMe and by Megalodon from PCR amplicons treated with M.SssI, pA-Hia5, or both. (c) Box plots showing the differences between the ONT reference model and the learned means from PCR amplicons treated with pA-Hia5. 6-mers were grouped based on the number of adenine(s) that they contained (6-mers without adenine n = 729, with 1 adenine n = 1,458, with 2 adenines n = 1,215, with 3 adenines n = 540, with 4 adenines n = 135, and with 5 adenines n = 18). Boxes extend from 25% to 75% quantiles, with the center denoting the median value. Whiskers extend to 1.5 times the interquartile range. The differences tend to be bigger for the *k*-mers with more adenine bases. Note the methylation efficiency of pA-Hia5 is very low at the sites with consecutive adenines. (d) Mean differences of *k*-mers with one adenine in different positions. (e) Mean differences of *k*-mers containing two consecutive adenines or two adenines separated by different number of nucleotides. B represents cytosine (C), guanine (G), and thymine (T). Source data are available in the Source Data file.

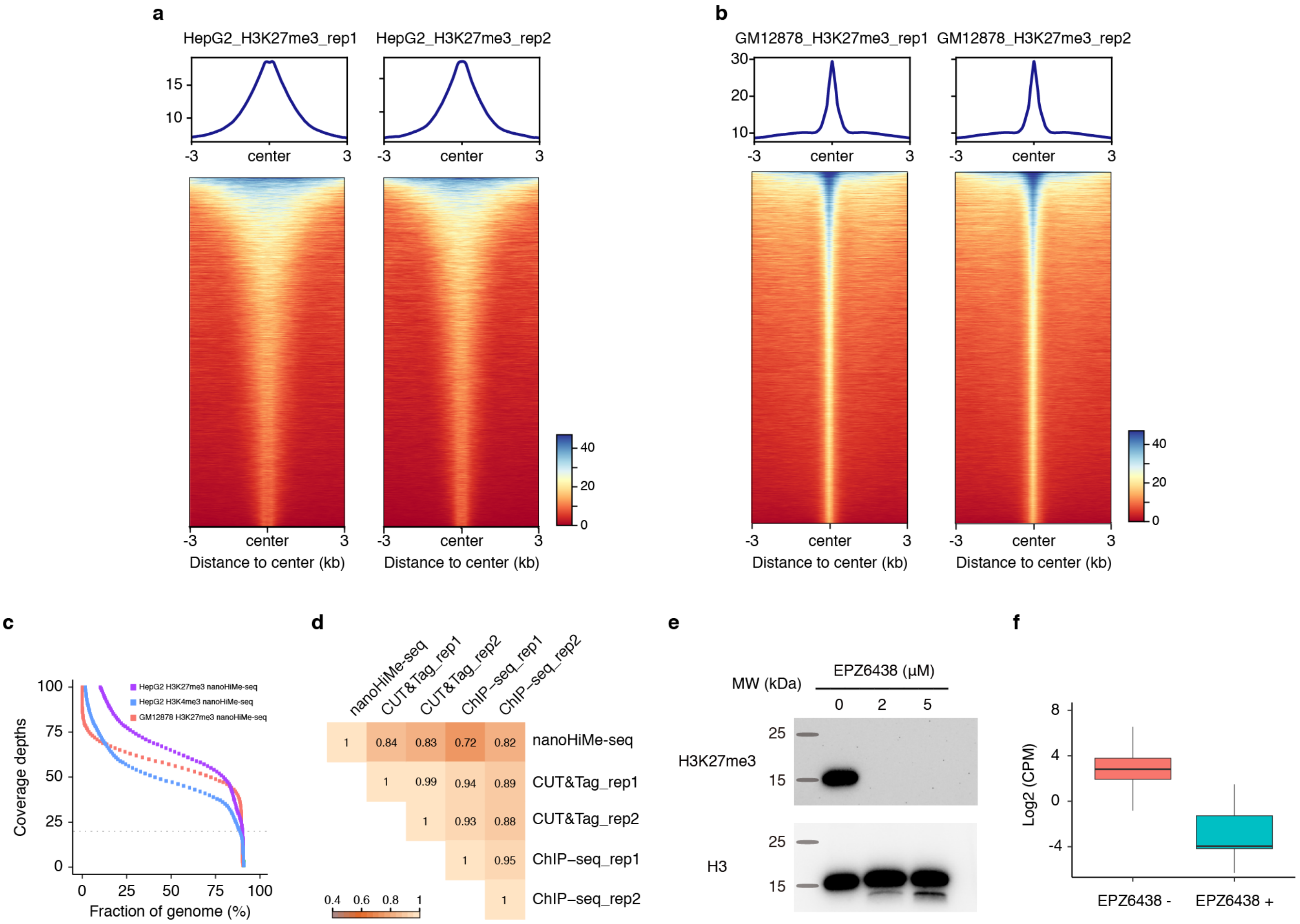
a
HepG2_H3K27me3_rep1
HepG2_H3K27me3_rep2
15
10
-3
center
3
Distance to center (kb)
40
20
0
b
GM12878_H3K27me3_rep1
GM12878_H3K27me3_rep2
30
20
10
-3
center
3
Distance to center (kb)
c
HepG2 H3K27me3 nanoHiMe-seq
HepG2 H3K4me3 nanoHiMe-seq
GM12878 H3K27me3 nanoHiMe-seq
Coverage depths
100
75
50
25
0
Fraction of genome (%)
d
nanoHiMe-seq
CUT&Tag_rep1
CUT&Tag_rep2
ChIP–seq_rep1
ChIP–seq_rep2
1
0.84
0.83
0.72
0.82
0.99
0.94
0.89
0.93
0.88
0.95
0.4
0.6
0.8
e
MW (kDa)
EPZ6438 (μM)
0
2
5
25
15
H3K27me3
H3
f
8
4
0
-4
Log2 (CPM)
EPZ6438 -
EPZ6438 +

Supplementary Figure 4. Profiles of H3K27me3 in HepG2 and GM12878 cells.

(a-b) Heatmaps showing H3K27me3 CUT&Tag signals around the center of peaks in HepG2 (a) and GM12878 (b) cells. The signals were sorted by the strength of enrichment (bottom). Average signals across peak center are also shown (top). (c) Coverage analysis of nanoHiMe-seq data for the indicated cells. The coverage depths were plotted as a function of the fraction of the reference genome. (d) Hierarchically clustered correlation matrix of nanoHiMe-seq, CUT&Tag and ChIP-seq profiling for H3K27me3 modification. Pearson correlations are calculated using read counts or the numbers of 6mA-containing sites across the top 50% peaks. (e) Western blots of nucleosomal histones from GM12878 cells treated with 0, 2 or 5 μM EZH2 inhibitor EPZ6438 for 7 days. Blots were probed with the indicated antibodies. (f) Box plots showing the 6mA signals from H3K27me3 nanoHiMe-seq in GM12878 cells without treatment or treated with 5 μM EZH2 inhibitor EPZ6438 for 7 days. The numbers of 6mA-containing sites in each of H3K27me3 CUT&Tag peaks (n=29613) were normalized as counts per million reads (CPM). Boxes represent median values, 25%, and 75% quantiles, and whiskers extend to 1.5 times the interquartile range. Source data are available in the Source Data file.

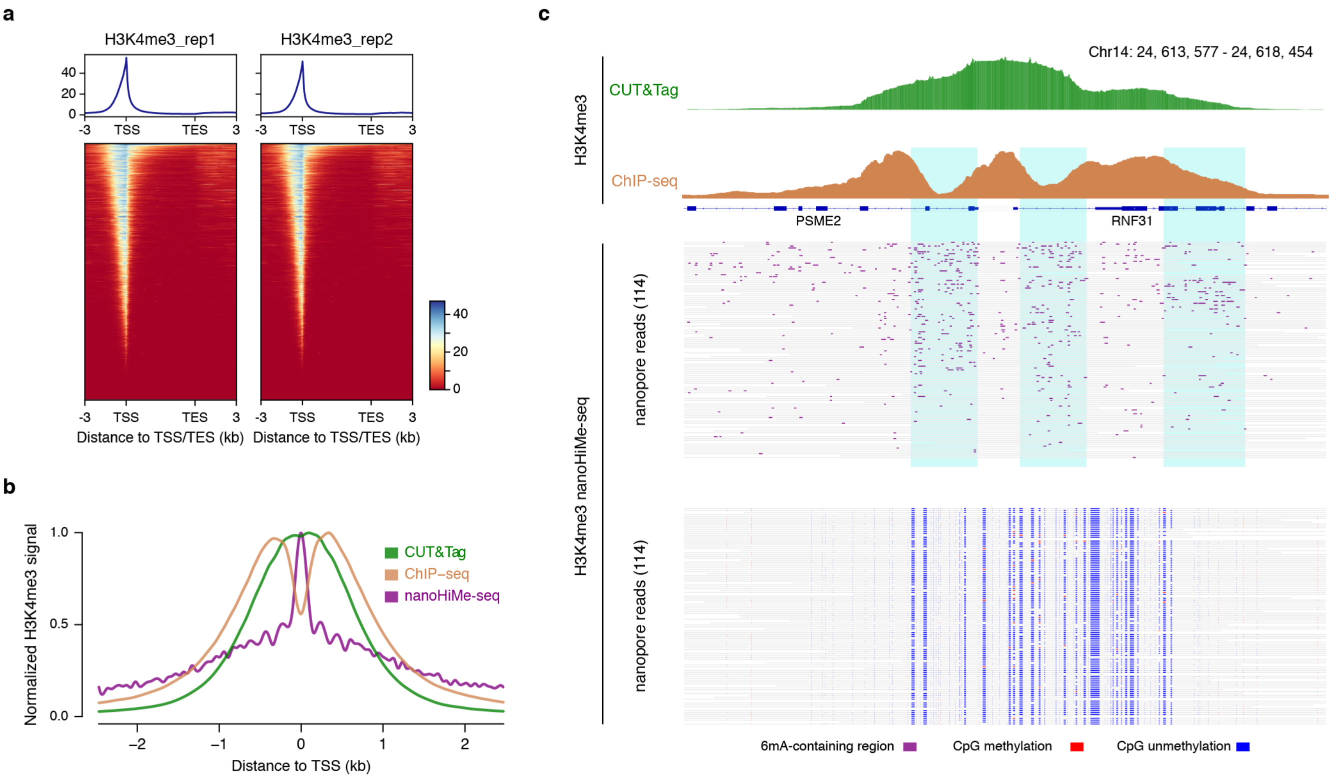

a
H3K4me3_rep1
H3K4me3_rep2
-3 TSS TES 3
Distance to TSS/TES (kb)
b
Normalized H3K4me3 signal
Distance to TSS (kb)
CUT&Tag
ChIP–seq
nanoHiMe-seq
c
Chr14: 24, 613, 577 - 24, 618, 454
H3K4me3
CUT&Tag
ChIP-seq
PSME2
RNF31
H3K4me3 nanoHiMe-seq
nanopore reads (114)
nanopore reads (114)
6mA-containing region
CpG methylation
CpG unmethylation

Supplementary Figure 5. Comparison of H3K4me3 profiles generated by nanoHiMe-seq, CUT&Tag, and ChIP-seq.

(a) Heatmaps showing H3K4me3 CUT&Tag results around all TSSs, sorted by the strength of enrichment signals (bottom). Average signals across all promoters were also shown (top). (b) Normalized average signals of H3K4me3 across active promoters. H3K4me3 enrichment determined by nanoHiMe-seq (purple), ChIP-seq (brown) and CUT&Tag (green) are shown. The signals from each method were computed for each 50-bp bin and then normalized to the maximum value. The active promoters were selected based on HepG2 RNA-seq data from ENCODE (see Methods) and the ones overlapping H3K4me3 peaks were used for the analysis. (c) A magnified view showing the calls of 6mA-containing sites (purple), methylated CpG sites (red) and unmethylated CpG sites (blue) on individual nanopore sequencing reads from H3K4me3 nanoHiMe-seq. Top panel shows enrichment signals from H3K4me3 CUT&Tag and ChIP-seq. The regions with high density of 6mA signals are shaded. Source data are available in the Source Data file.

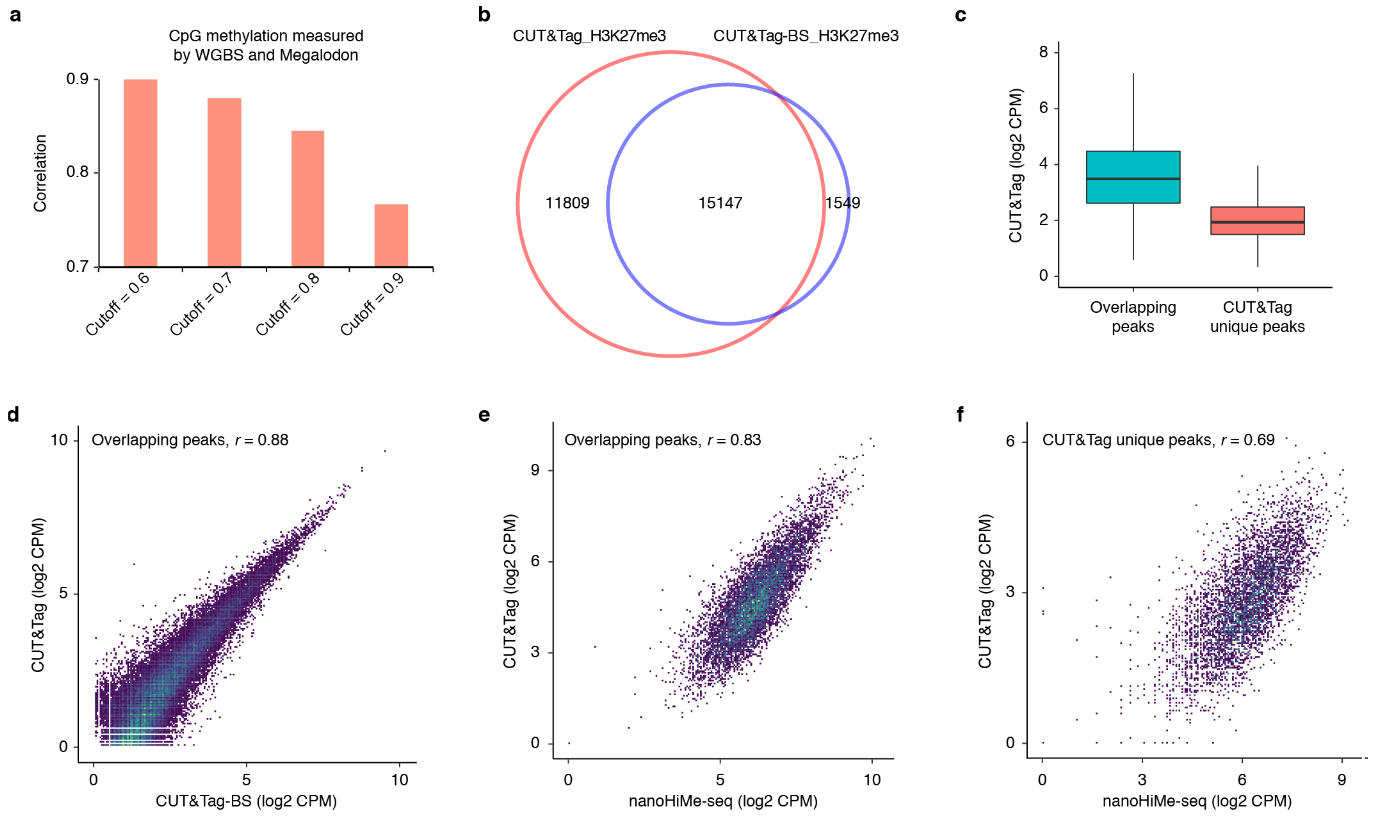

a
CpG methylation measured by WGBS and Megalodon
Correlation
0.9
0.8
0.7
Cutoff = 0.6
Cutoff = 0.7
Cutoff = 0.8
Cutoff = 0.9
b
CUT&Tag_H3K27me3
CUT&Tag-BS_H3K27me3
11809
15147
1549
c
CUT&Tag (log2 CPM)
8
6
4
2
0
Overlapping peaks
CUT&Tag unique peaks
d
Overlapping peaks, r = 0.88
CUT&Tag (log2 CPM)
CUT&Tag-BS (log2 CPM)
10
5
0
e
Overlapping peaks, r = 0.83
CUT&Tag (log2 CPM)
nanoHiMe-seq (log2 CPM)
9
6
3
0
f
CUT&Tag unique peaks, r = 0.69
CUT&Tag (log2 CPM)
nanoHiMe-seq (log2 CPM)
6
3
0
9

Supplementary Figure 6. Comparison of the performance of CUT&Tag, CUT&Tag-BS and nanoHiMe-seq for profiling H3K27me3.

(a) The Pearson correlation coefficients showing the comparison of CpG methylation levels measured by WGBS and by Megalodon using different cut-offs (from 0.6 to 0.9). (b) Venn diagram showing overlap of H3K27me3 peaks detected by CUT&Tag-BS and CUT&Tag. (c) Box plots showing H3K27me3 enrichment determined by CUT&Tag at peaks that were either recaptured (n=15147) or missed (n=11809) by CUT&Tag-BS. The signals in individual peaks were normalized as counts per million reads (CPM). Boxes represent median values, 25%, and 75% quantiles, and whiskers extend to 1.5 times the interquartile range.

(d) Density scatterplot showing correlation of H3K27me3 enrichment between CUT&Tag-BS and CUT&tag, with viridis color scale indicating density. Pearson's *r* value is shown at the top of each plot. (e-f) Density scatterplots showing correlation of H3K27me3 signals determined by CUT&Tag and nanoHiMe-seq at peaks that were either recaptured (e) or missed by CUT&Tag-BS (f). Source data are available in the Source Data file.

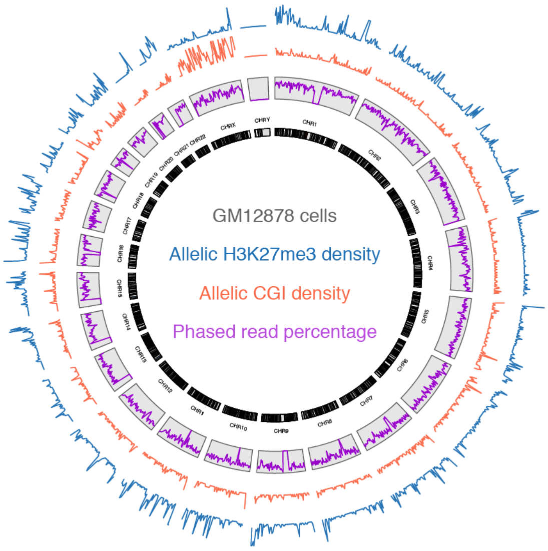
GM12878 cells
Allelic H3K27me3 density
Allelic CGI density
Phased read percentage

Supplementary Figure 7. Genome-wide distribution of allelic CpG islands (CGIs) and H3K27me3 in GM12878 cells.

Circos diagram of the genome showing the density of allelic CGIs (red) and H3K27me3 (blue) in moving 2 Mb windows (arbitrary scale). The percentages of nanopore reads (purple) that were phased in moving windows is also shown inside gray boxes. Source data are available in the Supplementary Data file 5-6.

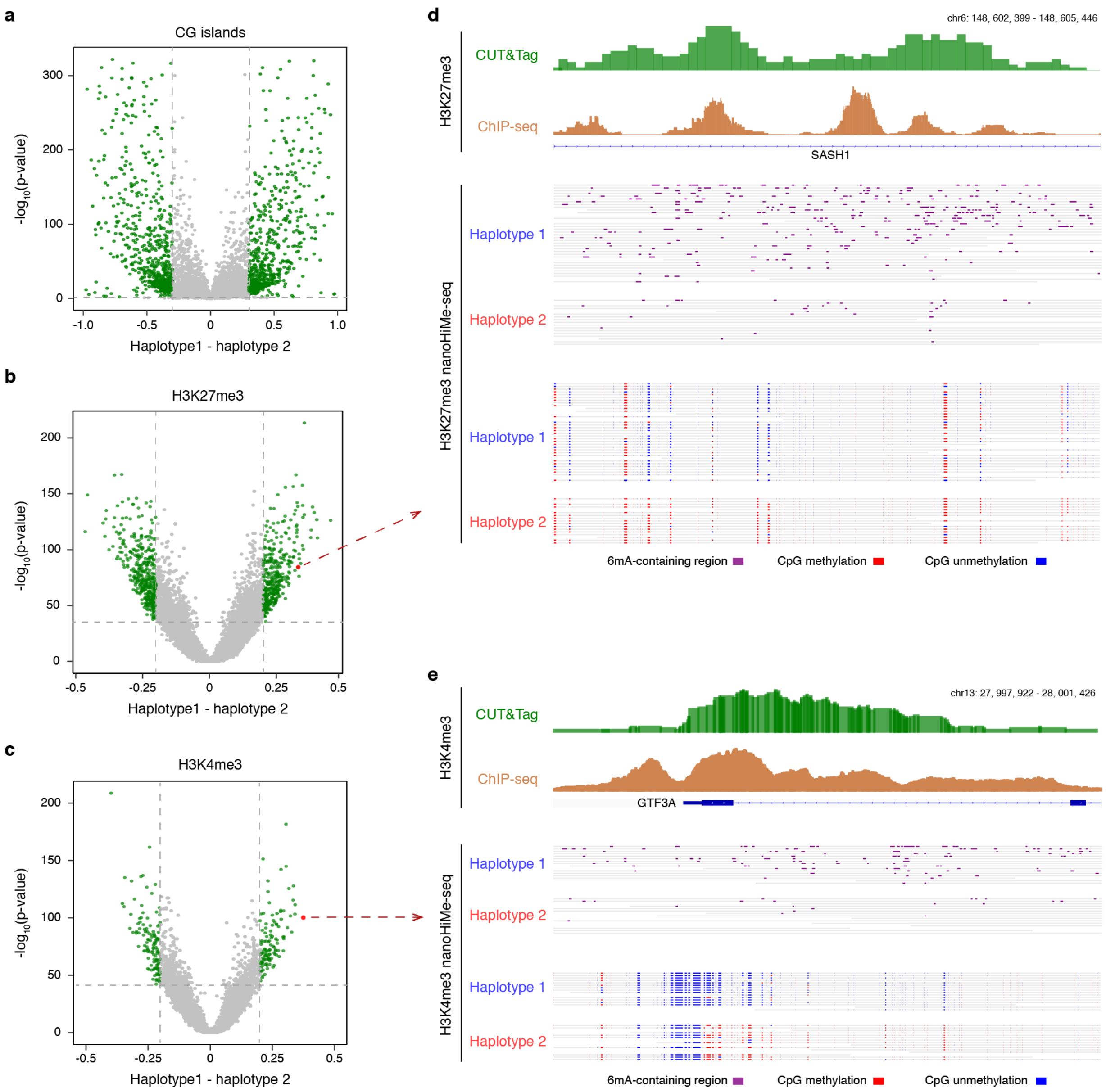
a
CG islands
-log10(p-value)
Haplotype1 - haplotype 2
b
H3K27me3
c
H3K4me3
d
chr6: 148, 602, 399 - 148, 605, 446
H3K27me3
CUT&Tag
ChIP-seq
SASH1
H3K27me3 nanoHiMe-seq
Haplotype 1
Haplotype 2
6mA-containing region
CpG methylation
CpG unmethylation
e
chr13: 27, 997, 922 - 28, 001, 426
H3K4me3
GTF3A
H3K4me3 nanoHiMe-seq

Supplementary Figure 8. Identification of allele-specific CpG methylation and histone modifications in HepG2 cells.

(a) Volcano plot of CGIs where ≥ 20 nanopore reads could be phased to respective haplotypes. CpG methylation levels were compared between two haplotypes for each CGI and the ones with differences ≥ 30% and p-value < 0.01(Fisher's exact test) were considered as allele-specific methylation regions (green). (b-c) Volcano plots of H3K27me3 (b) or H3K4me3 (c) peaks with dashed lines representing the thresholds (differences ≥ 20% and p-value < 0.05) for defining allelic histone modifications based on the allele-specific enrichment of 6mA signals. (d-e) Examples of a genomic region exhibiting allele-specific H3K27me3 (d) or H3K4me3 (e). Top panel shows H3K27me3 or H3K4me3 enrichment determined by CUT&Tag (green) and ChIP-seq (brown); Bottom panel shows the calls of 6mA-containing sites (purple), methylated CpG sites (red), and unmethylated CpG sites (blue) on individual phased nanopore reads from H3K27me3 or H3K4me3 nanoHiMe-seq. Statistical significance was calculated with two-sided Fisher's exact test, assuming equal variance and corrected for multiple hypotheses using Benjamini-Hochberg approach. Source data are available in the Supplementary Data file 5-7.

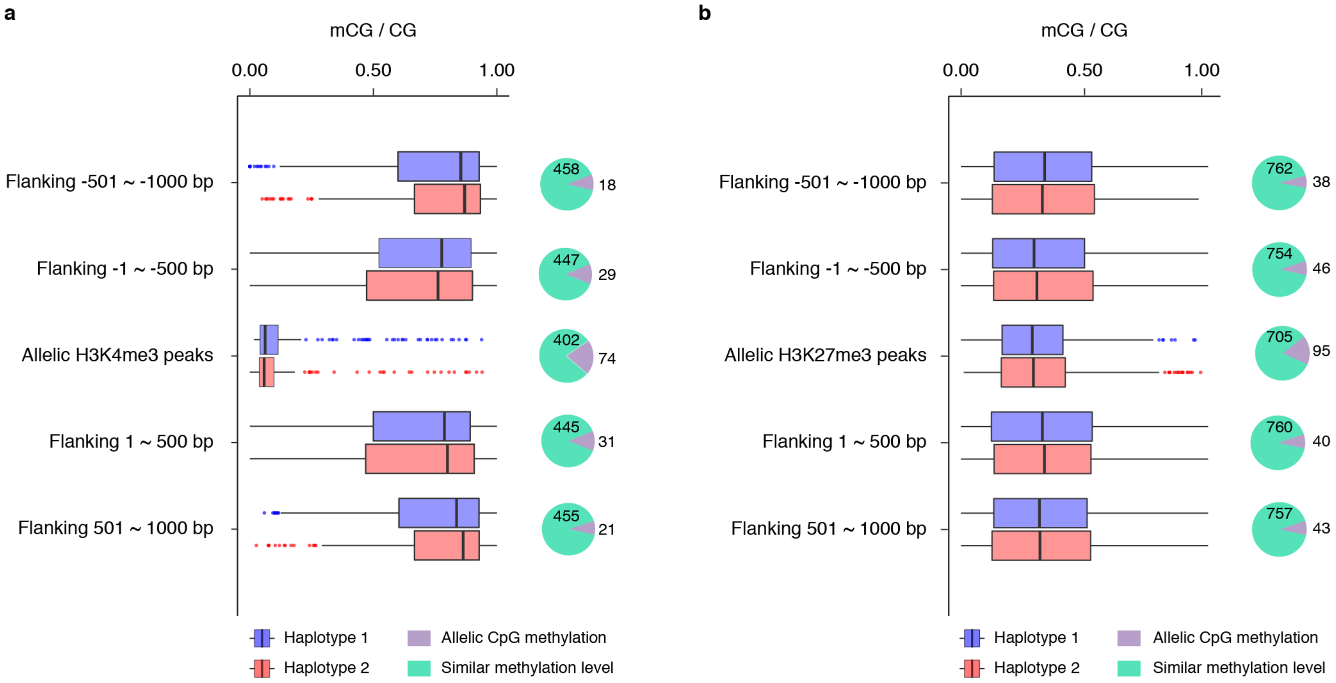
a
mCG / CG
0.00
0.50
1.00
Flanking -501 ~ -1000 bp
Flanking -1 ~ -500 bp
Allelic H3K4me3 peaks
Flanking 1 ~ 500 bp
Flanking 501 ~ 1000 bp
458
18
447
29
402
74
445
31
455
21
Haplotype 1
Haplotype 2
Allelic CpG methylation
Similar methylation level
b
mCG / CG
0.00
0.50
1.00
Flanking -501 ~ -1000 bp
Flanking -1 ~ -500 bp
Allelic H3K27me3 peaks
Flanking 1 ~ 500 bp
Flanking 501 ~ 1000 bp
762
38
754
46
705
95
760
40
757
43
Haplotype 1
Haplotype 2
Allelic CpG methylation
Similar methylation level

Supplementary Figure 9. Analysis of the correlation between CpG methylation and histone modifications in HepG2 cells.

(a-b) Analysis of the allelic CpG methylation within and outside (± 1 kb) of the peaks that exhibit allele-specific enrichment of H3K4me3 (n=476) or H3K27me3 (n=800). Left panel shows the CpG methylation within and outside of the peaks for each allele (haplotype 1 colored by blue and haplotype 2 by red) and right panel shows the number of regions that exhibit allele-specific CpG methylation (purple) or similar methylation level between the two haplotypes (cyan). Boxes represent median values, 25%, and 75% quantiles, whiskers extend to 1.5 times the interquartile range, and discrete points represent outliers. Source data are available in the Supplementary Data file 5-7.
