# Supplementary material for: Simultaneous profiling of histone modifications and DNA methylation via nanopore sequencing
Source: Nat Commun. 2022 Dec 24;13:7939. doi: 10.1038/s41467-022-35650-2 (PMC9789962; doi:10.1038/s41467-022-35650-2)
Supplement: Supplementary file 3 — Description of additional Supplementary File [file 41467_2022_35650_MOESM3_ESM.pdf]

# **Descriptions of additional supplementary files**

Supplementary Data 1: The table contains DNA sequences used in vitro methyltransferase activity assessment, CUT&Tag and CUT&Tag-BS

Supplementary Data 2: Statistics of nanoHiMe-seq sequencing data and the datasets for model training and assessment

Supplementary Data 3: Contingency table for the positive and negative control accuracy assessment

Supplementary Data 4: Phasing quality assessment

Supplementary Data 5: Allele-specific CG islands in HepG2 and GM12878 cells

Supplementary Data 6: Allele-specific H3K27me3 peaks in HepG2 and GM12878 cells

Supplementary Data 7: Allele-specific H3K4me3 peaks in HepG2 cells
